# Lipschutz’s vulvar ulcer in an adolescent after Pifzer COVID-19 vaccine^☆^

**DOI:** 10.1016/j.abd.2023.03.003

**Published:** 2023-08-29

**Authors:** Juan-Manuel Morón-Ocaña, Ana-Isabel Lorente-Lavirgen, Isabel-María Coronel-Pérez, María-Luisa Martínez-Barranca

**Affiliations:** Department of Dermatology, Hospital Universitario Virgen de Valme, Sevilla, Spain

*Dear Editor,*

Lipschütz’s acute vulvar ulceration is a non-sexually acquired condition, which is characterized by a sudden onset of a few necrotic and painful genital ulcers. Self-resolution without scarring and relapses is the usual course.^1^ The underlying pathogenesis of vulvar aphthous ulcers is unclear. Numerous case reports have described aphthous ulcers as a dysregulated immune response associated with a variety of infections including Cytomegalovirus (CMV), influenza, mumps virus, salmonella, mycoplasma and most notably Epstein Barr Virus (EBV).^2^

Over 334,000,000 doses of the Moderna, Pfizer and Johnson & Johnson vaccines have been administered since December 2020. Side effects are common and have been widely reported. Most of the systemic side effects after receiving the Pfizer COVID-19 vaccine as headache, fatigue, chills, diarrhea, fever and myalgias are well-known.^3^ However, skin manifestations are not as well studied.^4^ In this brief report, the authors present the case of a patient presenting with vulvar aphthous ulcer following Pfizer COVID-19 vaccination.

A 13-year-old girl, with no relevant personal history, started with fever, myalgias, and intense pain in her genitals 48 hours after having been vaccinated with the second dose of the COVID-19 vaccine (Pfizer).

After being explored, fibrinous kissing ulcers were observed on the vulva (Fig. 1).

**Figure 1:**
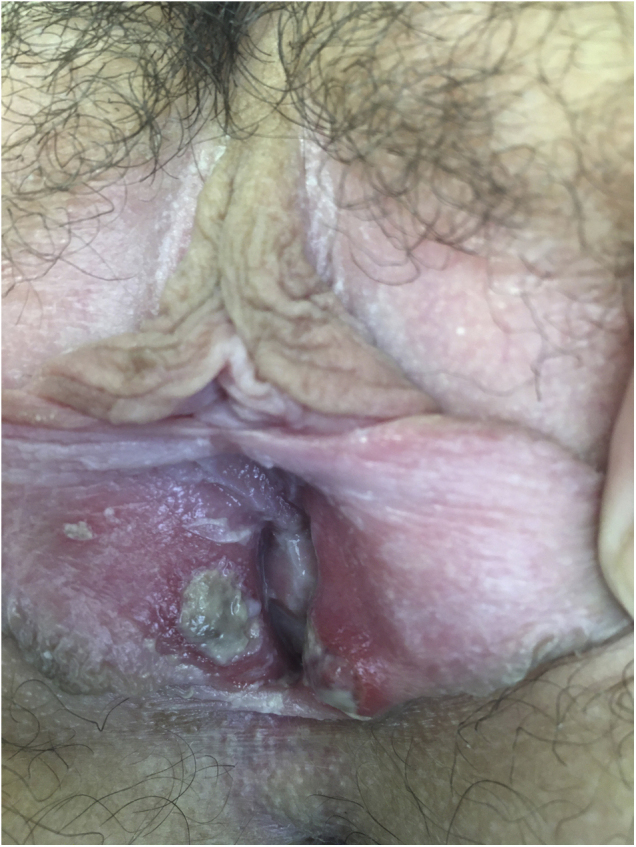
Fibrinous kissing ulcers on the vulva.

The patient denied initiation of sexual intercourse and had had the menarche a year ago with regular menstruations that did not coincide with the ulcers.

Exudated cultures and PCRs for herpes viruses 1 and 2, treponema, and mycoplasma of the vulva were negative. Serological tests including HIV, Epstein Barr virus and antinuclear antibodies were also negative. The patient also presented a negative nasopharyngeal test for SARS-CoV-2 infection.

Empirical treatment with topical antibiotics and corticosteroids was started. In 15 days, the patient was reevaluated with a complete resolution of the injuries.

The case was reported to the Spanish pharmacovigilance system. According to its casualty algorithm, the vulvar ulcer was considered as possibly related to the vaccine.

One of the rare side effects of the COVID-19 vaccine may be the appearance of ulcers on the vulva. In fact, among the 1,128,289 cases of adverse effects by the Pfizer COVID-19 vaccine that the pharmacovigilance database of the European Medicine Agency (eudravigilance) has reported up to 24/09/22; 47 cases were vulvar ulcers.^5^

If the authors compare with other vaccines against COVID-19 disease, 9 cases of vulvar ulcers have been described with the Moderna vaccine, 19 with the Astrazeneca vaccine and 1 with the Janssen vaccine up to 24/09/22.^5^

This is one case report to describe a potential relationship between the development of vulvar aphthous ulcers and COVID-19 vaccination. Our patient had typical clinical features of aphthous ulcer including an influenza-like prodrome and characteristic dermatologic manifestations which occurred after receiving the second dose Pfizer COVID vaccine.^6^

Vulvar aphthous ulcers are thought to be precipitated by physiologic stress from a variety of insults, most notably viral infections. The Pfizer BioNTech (BNT162b2) COVID-19 vaccine, which our patient received, is a lipid nanoparticle-formulated nucleoside-modified mRNA that encodes the receptor binding domain of the SARS-CoV-2 spike glycoprotein. The spike glycoprotein is a popular target in COVID-19 vaccine development as it mediates the entry of SARS-CoV-2 into host cells by binding the angiotensin-converting enzyme 2 receptor.^7^

The temporal relationship of our patient’s symptoms to Pfizer BioNTech (BNT162b2) vaccination in the context of a negative test for SARS-COV-2 RNA, as well as other infectious etiologies, suggest that her systemic symptoms and vulvar aphthous ulcer may have occurred secondary to an immune response precipitated by the vaccine.

In summary, this case highlights a potential novel association between mRNA Pfizer BioNTech (BNT162b2) COVID-19 vaccination and vulvar aphthous ulcer. One proposed mechanism for the study is to investigate how the immune system’s response to vaccination recapitulates the pro-inflammatory response associated with vulvar aphthous ulcers secondary to a viral illness.

In the case of our patient, the immune response could be triggered by the virus proteins of the Pfizer COVID-19 vaccine after no other underlying cause was found.

## Financial support

None declared.

## Author's contribution

Juan Manuel Morón Ocaña: Preparation and writing of the manuscript; critical literature review.

Ana Isabel Lorente Lavirgen: Approval of the final version of the manuscript; manuscript critical review.

Isabel María Coronel Pérez: Approval of the final version of the manuscript; manuscript critical review.

María Luisa Martínez Barranca: Approval of the final version of the manuscript; manuscript critical review.

## Conflicts of interest

None declared.
